# Impact of cataract on the spectral measurement of fundus autofluorescence

**DOI:** 10.1007/s00417-022-05554-4

**Published:** 2022-01-12

**Authors:** Rowena Simon, Jakob Lauritz Brauer, Daniel Meller, Martin Hammer

**Affiliations:** 1 Department of Ophthalmology, University Hospital Jena, Am Klinikum 1, 07747 Jena, Germany; 2 Center for Medical Optics and Photonics, Univ. of Jena, Jena, Germany


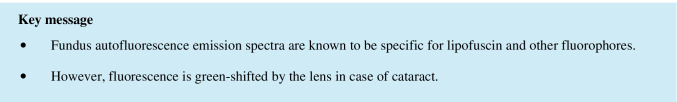


Dear Editor,

Fundus autofluorescence (FAF) can arise from a variety of fluorophores. To distinguish those fluorophores, FAF can be characterized by its fluorescence lifetime and by its emission spectrum. The spectral characteristic of FAF can give valuable information on pathologic changes in RPE and accumulation of metabolic byproducts in age-related macular degeneration [1, 2]. Recently, we introduced the peak emission wavelength (PEW), calculated from the ratio of fluorescence intensities in the two spectral channels of the fluorescence lifetime imaging ophthalmoscope (FLIO), as an estimate measure of the emission spectrum [3]. Here, we investigate the influence of cataract on the PEW.

We performed FLIO measurements of FAF in 32 eyes of 32 patients (age: 72.3 ± 9.3 years) scheduled for cataract extraction. The study followed the tenets of the Declaration of Helsinki and was approved by a local ethics committee. Informed consent was obtained from all subjects before inclusion. FLIO investigations were performed one day before and on average 33.2 (± 10.3) days after cataract extraction. FLIO counts single fluorescence photons with respect to their time delay relative to the excitation laser pulse (470 nm) in two spectral channels (500–560 nm and 560–720 nm). Here we disregard the temporal information and calculate the PEW from the ratio of fluorescence photons per pixel in the two spectral channels as described earlier [3]. PEW were averaged over standardized areas at the fundus by centering the standard ETDRS grid at the fovea. The foveal PEW was calculated as mean value of pixels within the center of the grid and the value for the posterior pole as average over the pixels in the inner and outer ring of the grid.

**Fig. 1:**
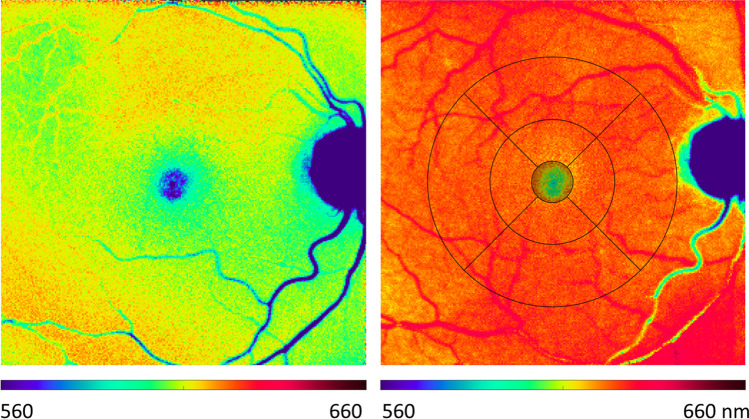
Color-coded PEW (see scale bar) of a patient before (left) and after (right) cataract extraction. In the right-hand panel, the ETDRS grid is shown

**Fig. 2:**
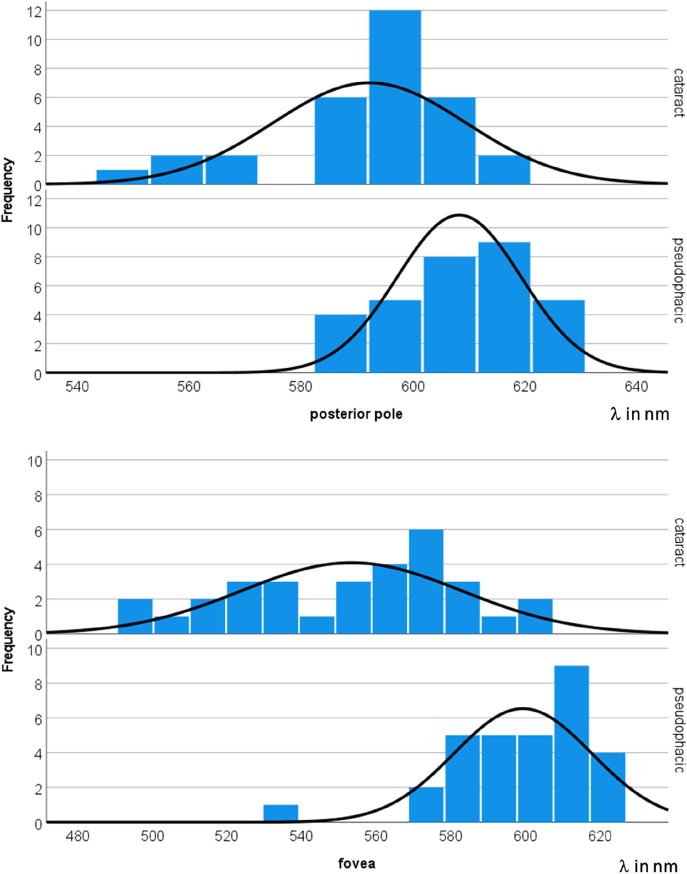
Histogram of PEW distributions at the posterior pole (top) and fovea (bottom) over all patients (n = 32) before and after cataract extraction

Figure 1 shows the color-coded PEW for one subject before (left) and after (right) cataract extraction; a red-shift is clearly seen. Averaged over all subjects, the PEWs were 523 ± 29 nm and 592 ± 17 nm before as well as 599 ± 18 nm and 608 ± 11 nm after cataract extraction in the fovea and at the posterior pole, respectively (Fig. 2). No difference between nuclear (*N* = 12) and cortical (*N* = 20) cataract was found for the pre-operative PEW. The pre- and post-operative values were different with high significance (*p* < 0.001 in the t-test in both areas) and the effect was strong (Cohen’s d = 1.416 for fovea and 0.723 for the posterior pole).

This indicates that FAF observations, even if using confocal scanning techniques, are overlaid by the lens fluorescence. This way, our current study corroborates similar observations with respect to fluorescence lifetime [4]. As the human lens has a fluorescence emission maximum at about 515 nm [5], its removal results in longer emission FAF wavelengths which are in agreement with that of lipofuscin [1]. The difference of PEW pre- and post-surgery is greater in the fovea than at the posterior pole. The reason might be the lower fluorescence intensity due to Xanthophyll absorption of the excitation light. This results in a higher relative contribution of the cataract lens to the total fluorescence in the fovea. In summary, our investigation shows that the fluorescence of the lens shifts the measured FAF toward shorter wavelength. Although this was shown in the current study for cataract lenses only, we assume that this held in general for aged lenses.
